# mHealth Intervention to Improve Treatment Outcomes Among People With HIV Who Use Cocaine: Protocol for a Pilot Randomized Controlled Trial

**DOI:** 10.2196/28332

**Published:** 2022-03-07

**Authors:** Yerina S Ranjit, Archana Krishnan, Debarchana Ghosh, Claire Cravero, Xin Zhou, Frederick L Altice

**Affiliations:** 1 Department of Communication University of Missouri Columbia, MO United States; 2 Department of Communication University at Albany State University of New York New York, NY United States; 3 Department of Geography University of Connecticut Storrs, CT United States; 4 Harvard T H Chan School of Public Health Harvard University Boston, MA United States; 5 Department of Internal Medicine, Section of Infectious Diseases Yale University New Haven, CT United States

**Keywords:** smart pillbox, smartphone, mHealth intervention, people with HIV, cocaine use, antiretroviral therapy, description of feasibility and acceptability, mobile phone

## Abstract

**Background:**

Antiretroviral therapy is effective in reducing HIV-related morbidity, mortality, and transmission among people with HIV. However, adherence and persistence to antiretroviral therapy are crucial for successful HIV treatment outcomes. People with HIV who use cocaine have poor access to HIV services and lower retention in care.

**Objective:**

The primary goal of this paper is to provide a detailed description of a mobile health intervention. This study is designed to improve medication adherence among people with HIV who use cocaine. A secondary goal is to list the important challenges and adaptations incorporated in the study design.

**Methods:**

This study, titled Project SMART, used a wireless technology–based intervention, including cellular-enabled electronic pillboxes called TowerView Health and smartphones, to provide reminders and feedback on adherence behavior. The intervention design was based on the theoretical frameworks provided by the self-determination theory and the Motivation Technology Model. The 12-week pilot randomized controlled trial with four arms provided three types of feedback: automated feedback, automated+clinician feedback, and automated feedback+social network feedback.

**Results:**

The study was funded by the National Institute of Drug Abuse (R21DA039842) on August 1, 2016. The institutional review board for the study was approved by Yale University on March 21, 2017. Data collection lasted from June 2017 to January 2020. The final enrollment was 71 participants, of whom 57 (80%) completed the study. The data are currently undergoing analysis, and the manuscript is being developed for publication in early 2022.

**Conclusions:**

Implementing complex mobile health interventions for high-risk and marginalized populations with multicomponent interventions poses certain challenges, such as finding companies with adequate technology for clients and financial stability and minimizing the research-related burden for the study population. Conducting feasibility studies is important to recognize these challenges and the opportunity to address these challenges with solutions while keeping the design of a randomized controlled trial as true as possible.

**Trial Registration:**

Clinicaltrials.gov NCT04418076; https://clinicaltrials.gov/ct2/show/NCT04418076

**International Registered Report Identifier (IRRID):**

DERR1-10.2196/28332

## Introduction

### Background

Antiretroviral therapy (ART), a daily medication regimen, is extremely effective in reducing HIV-related morbidity and mortality and reducing transmission among people with HIV [1-3]. ART suppresses HIV viral load (VL) and slows the progression of the virus in the body by keeping a high CD4 cell count [4]. The Centers for Disease Control and Prevention defines viral suppression as having <200 copies of HIV per milliliter of blood [4]. However, successful HIV treatment outcomes depend on patients’ adherence to and persistence in ART [5-9]. When adherence is optimal (≥90%) [10], improvements are seen not only for HIV outcomes but also for several comorbidities, including substance use disorders, tuberculosis, viral hepatitis, and depression [11-15]. Among the approximately 1.2 million people with HIV in the United States, nonadherence to ART results in as few as 19% achieving viral suppression [16]. For people with HIV with co-occurring substance use disorders, there are additional negative health outcomes. Active drug use is associated with clinicians not prescribing or delaying prescribing ART [17], which can overshadow a patient’s health care priorities, including medication adherence and retention in HIV care [18]. As a result, people with HIV who use drugs experience worse clinical outcomes and greater mortality than people who do not use drugs [19]. People who use cocaine especially have poor measures of success in accessing and retaining HIV care [20,21]. They are less likely to receive ART compared with people who use other drugs [21,22], who have poor adherence to ART when they do receive it [23-25], and who have an extraordinarily low likelihood of achieving viral suppression [26]. Overall, AIDS-related death rates among people who use cocaine are 3 times higher than among those who do not use cocaine, controlling for age, race, duration of illness, and self-reported high ART adherence [26]. Effective strategies for improving ART adherence are urgently needed to address this health crisis among people with HIV who use cocaine.

For people with HIV who use cocaine, currently, the only effective adherence intervention is directly administered ART, which is expensive and labor intensive [27]. International guidelines suggest that adherence interventions need to be scaled back, both in terms of cost and personnel [28]. Mobile health (mHealth) technologies satisfy these recommendations and can provide innovative, efficacious, and cost-effective strategies for improving ART adherence and optimizing HIV treatment outcomes [29,30]. In fact, the World Health Organization guidelines now strongly recommend sending SMS text messages to promote adherence and retention in general [31]. mHealth has already shown promise in resource-limited settings [32] and improvement in treatment efficacy among patients with diabetes [33,34], tuberculosis [35], malaria [36], asthma [37], and HIV [38-40]. mHealth interventions have also been efficacious in reducing substance use [41]. In addition, bidirectional SMS text messaging interventions have significantly improved adherence to ART among people with HIV who use substances [42]. However, there has *never* been any systematic development or testing of mHealth interventions for people with HIV who use cocaine. Moreover, existing mHealth interventions have not optimized the integration of feedback to determine what type of feedback (eg, predetermined by researchers or clinicians, tailored to patients’ preferences, dynamically tailored based on patient’s adherence level, or feedback from friends and family members) improves outcomes. Prior studies have shown the effects of dynamically tailored feedback via SMS text messages on viral suppression compared with standard HIV care [43-45]. However, in these studies, the feedback was designed for people with HIV in a regulated clinical setting, which may not be effective for people with HIV using drugs in an unregulated community setting.

### Study Objective

The primary objective of this paper is to provide a detailed description of a pilot mHealth-based randomized controlled trial (RCT) for improving ART adherence among people with HIV who use cocaine. A secondary goal is to list the critical challenges faced during the duration of the study and adaptations incorporated into the study design.

Pilot RCTs are small-scale studies designed to evaluate the feasibility and acceptability of a proposed intervention. Findings related to feasibility prepare investigators to conduct large-scale interventions, whereas acceptability shows how the population for whom the intervention is designed and the researchers who implement the study react to the intervention [46]. As part of a National Institute on Drug Abuse–funded study, we developed and conducted a feasibility study titled Project SMART, which developed a wireless technology–based intervention with multiple forms of feedback to improve ART adherence. In this paper, we aim to describe the research design of this pilot study, including the timeline, assessments, technologies, and challenges faced by researchers in implementing a multicomponent intervention. We also outline some practical adjustments made to the protocol during the study. By sharing our experiences, we aim to inform and educate other researchers interested in designing mHealth interventions for populations with similar characteristics. The following sections describe the methodologies and components of the pilot study as these are preanalysis protocol descriptions. The primary and secondary outcomes will be analyzed in forthcoming papers.

## Methods

### Study Setting

This study was conducted between May 2017 and January 2020. Participants were recruited from community centers, HIV care clinics, and a mobile clinic that has strategic liaisons with patients and providers throughout the Yale University community. The study took place within an HIV and opioid use disorder treatment facility, which is in an urban location in the northeastern part of the United States. The study was funded by the National Institute of Drug Abuse (R21DA039842).

### Recruitment, Screening for Eligibility, and Consent

Recruitment started in May 2017 and continued until January 2020. Institutional review board (IRB)-approved flyers containing a toll-free number were posted in numerous venues such as community centers, HIV care clinics, and a mobile clinic that had strategic liaisons with patients and providers throughout the community. The initial screening eligibility criteria included patients (1) with a self-reported HIV diagnosis, (2) with self-reported use of cocaine ever, (3) having ART prescription and taking ART, and (4) willing and able to use a smartphone and electronic pillbox for a 16-week study period. After screening, the research staff obtained informed consent from the participants at their first visit and explained the purpose, protocol, expectations, and possible risks and benefits of the study.

### Study Design

The design of this study is based on the fundamental components of transactional communication, which are the sender, receiver, and message [47]. In the transactional model of face-to-face communication, the sender and receiver engage in communication facilitated by feedback (verbal or nonverbal). With technologies such as the electronic pillbox, this exchange can take place only when the technology is responsive in some form to its user [48]. Interactive technologies enhance the engagement of its users [48]. Self-determination theory posits that the motivation to perform an action is influenced if an individual’s need for competence, autonomy, and relatedness is fulfilled [49]. Using this assumption, the Motivation Technology Model (MTM) predicts that individuals can be motivated to change their behaviors if and when they achieve a sense of relatedness while using the technology [50]. MTM argues that this interactive nature of technology, which fulfills the need to be socially connected, can motivate individuals to perform or maintain certain health behaviors [50]. This perception of interactivity can be established when the technology provides timely responses and feedback to its users [50]. On the basis of these theoretical concepts of the self-determination theory and MTM, this study uses feedback as the central motivational factor to study adherence behavior.

**Figure 1:**
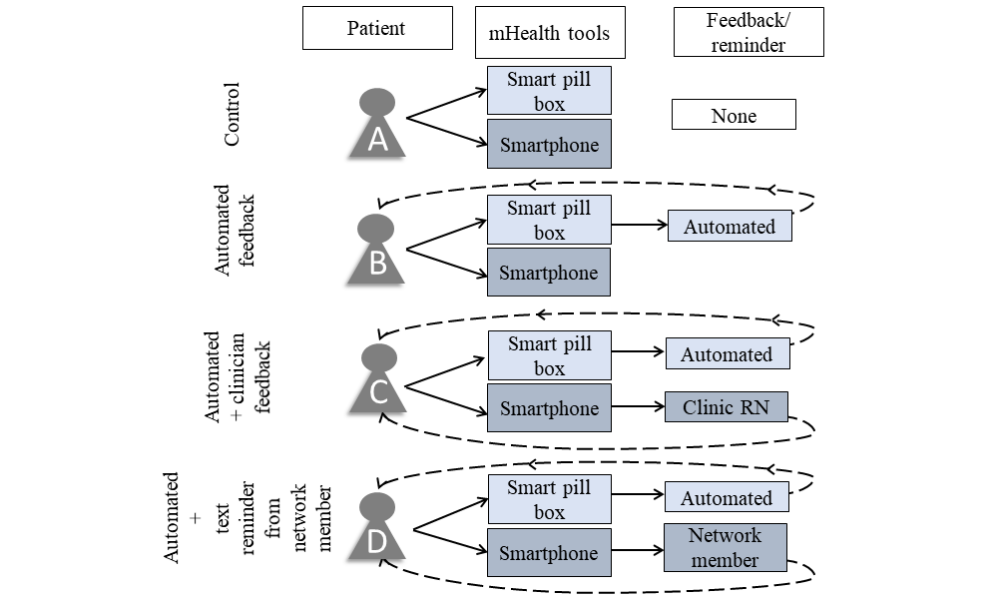
Study design of Project SMART. RN: registered nurse.

The study design of Project SMART is shown in Figure 1. This was a 12-week pilot RCT that examined the use of two mHealth tools (cellular-enabled electronic pillboxes and smartphones) and four types of feedback—(1) no feedback, (2) automated feedback, (3) automated+clinician feedback, and (4) automated feedback+social network feedback (Figure 1)—on primary (ART adherence and persistence) and secondary outcomes (HIV viral suppression, cocaine use, retention in HIV care, and retention in RCT). The study period was 16 weeks, with a 12-week intervention period and a 4-week postintervention period. The following sections describe each of the components of the study design in detail.

### mHealth Tools

#### Overview

Two mHealth tools—an electronic pillbox and smartphones—were used for intervention delivery. All study participants received these two mHealth tools but differed in the type of communication feedback they received: no feedback, automated feedback, automated and clinician feedback, and automated and social network feedback. The types of feedback determined the four arms of the RCT: 1 control arm and 3 intervention arms.

#### Electronic Pillbox

##### Overview

**Figure 2:**
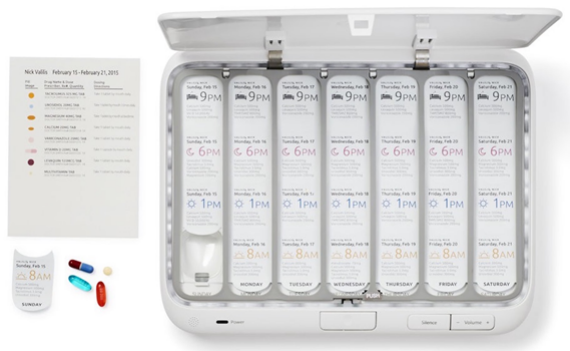
Electronic pillbox TowerView Health used for Project SMART.

As shown in Figure 2, the TowerView Health pillbox is 10×7.5×1.5 inches, which is comparable with a handheld device such as an iPad, and can be linked to a smartphone. It has the capacity to hold pills or tablets for a weekly medication regimen of up to 4 dosages a day. The pills are nestled in 28 individual wells, each with its own sensor, to indicate when a dosage has been removed. The box has a built-in alarm that emits both a low sound and a flash of light. The pillboxes have 4G cellular capability via an integrated chip and hence have the ability to communicate with other devices such as smartphones. This capability is useful for transmitting real-time adherence data to the secure TowerView Health server and communicating with linked smartphones via SMS text messages.

##### Medication and Refills

At the start of study enrollment, the clinician associated with Project SMART requested permission from participants’ physicians to dispense ART medications from the selected pharmacy for the study period. The pharmacy collaborated with TowerView Health and delivered 4 sets (one for each week) of blister packs, with 1 months’ worth of ART medication specifically designed to fit the dimensions of the pillbox. Although the pillboxes are designed to hold medication pills independently, blister packs were used by TowerView Health for ease of delivery and organization as they did not require manual insertion of individual pills. Our clinical staff placed these blister packs in the box in the participants’ presence for the first time and trained the participants to replace the blister packs at the end of each week. Participants were instructed to bring their pillbox to the research site each month, at which time the research staff resupplied participants with a month’s supply of medications. A 1-month supply of medication is the community standard of care for the dispensation of medications for patients with HIV [51].

##### Automated Feedback Messages

All participants’ phones were connected to their assigned pillbox. The pillbox was programmed to send SMS text messages in the event of a missed dosage or messages of encouragement in the event of a successful daily dosage regimen. For example, when the pillbox was opened, and a segment of the blister pack was broken for medication intake, the sensor within the individual wells detected pressure, prompting a signal to be sent to the TowerView Health server. This then activated the automatic delivery of an SMS text message to the participant’s smartphone. The content of the SMS text messages or feedback delivered to a participant’s smartphone was determined by the research team. Refer to section *Randomization Trial and Intervention* for examples of messages sent to participants.

#### Smartphones

The study used Samsung phones with an Android operating system, which were purchased through Yale University’s agreement with Verizon. The participants received either a Samsung 7 or Samsung 8 model for the duration of the study, and the phone was configured for our study. The messaging app was activated so that participants could start receiving SMS text messages from the electronic pillbox. A total of 2 apps, *CommCare* and *SMS Back-Up and Restore*, were installed before handing out a phone to a participant (the role of these apps is described in the *Study Instruments* section). The research staff trained the participants on how to charge and use the electronic pillbox and smartphone (including using the voicemail and SMS text messaging features). In addition, the research staff programmed pertinent contact information (eg, clinic care and research staff contact) on each participant’s phone.

### Randomization Trial and Intervention

After completing the baseline interview, participants were assigned to 1 of the 4 arms systematically at a 1:1:1:1 ratio. Arm A was the control group, and participants in this group did not receive feedback as an intervention via the mHealth tools. The in-built and automatic feedback features of the TowerView Health smart pillboxes were also deactivated.

Arm B participants received automated feedback messages as an intervention. The box sent text dosage reminders to the participants’ phones at the dosage time, and the rim around the box flashed blue, purple, and magenta light-emitting diode lights with a gentle ringing alarm. An example of the SMS text message is as follows: “Hi [participant name], don’t forget to take your [dose time, eg, 3 PM] medications.” If the pillbox was not opened at a dosage time, the box sent a reminder at 30 minutes and another at 90 minutes past the missed dosage time. The reminder said: “Hello [participant name], here’s a quick reminder to take your missed pill of [dose time].” If at the end of the day, the participant took all the dosages, they received a note of encouragement on their phones; for example, “Congratulations, [participant name]! You took all your pills yesterday*.*” These kinds of positive feedback were accompanied by emoticons such as a thumbs-up or clap. The positive messages varied daily and were selected from a set of messages designed by the team before the start of the study.

Arm C participants, in addition to the automated feedback from the pillbox, received feedback about their ART adherence from a clinician via phone calls on a weekly basis. The clinician’s phone conversation was guided by the real-time adherence data provided by the TowerView Health dashboard.

For arm D, in addition to the automated feedback from the pillbox, participants received SMS text message reminders to take their medication from a person in their social network or a preselected social designee. The designee was a family member, friend, or acquaintance (excluding clinicians) who was willing to be part of the intervention and consented to their participation. The research team sent a weekly notification to the designee’s mobile phone, prompting them to deliver the intervention via SMS text messages to the participant. The predefined automated notification was:

This is from Project SMART. In the next hour, send a text to [participant’ name] @ [participant’ number] to remind them to take their medication on time. Thank you for your help.!

The designee then sent SMS text messages to remind the participant’s phone about their ART dosages. No predefined SMS text messages for dyads (participant–social designee) were developed for this intervention. Instead, the actual contents of the SMS text message reminders between dyads were recorded as intervention data.

Participants in arms B, C, and D received their interventions for a duration of 12 weeks. After 12 weeks, the built-in feedback features of the electronic pillbox were deactivated such that during the 4-week postintervention period, the pillboxes only measured participants’ pill-taking behavior, similar to arm A.

### Study Instruments

#### Overview

Project SMART collected data using a range of instruments and standardized assessments administered at various time points of a participant’s visits during preintervention (screening and baseline), intervention (4, 8, and 12 weeks), and postintervention (16 weeks) phases, spanning a period of 16 weeks. Table 1 contains a list of survey instruments.

**Table 1:** Study activity and instruments.

Study activity and measures		Preintervention			Intervention				Postintervention, week 16
		Before 4 weeks to before 1 week	Baseline (enrollment)	Preparation time	Week 0	Week 4	Week 8	Week 12	
**Study activity**									
	Screening for eligibility	✓							
	Release of information		✓						
	Randomization			✓					
	Device feature setting and preparation			✓					
	Acquiring permission to change pharmacy and ordering medication			✓					
	Device and medication distribution				✓				
	Assigning social network designee		✓						
	Phlebotomy		✓					✓	
	Weekly smartphone survey (conducted weekly)					✓	✓	✓	✓
**Demographic and social information**									
	Demographics		✓						
	Social circumstances		✓			✓	✓	✓	✓
	Health care status								
	Health literacy [52]		✓						✓
	Social support [52]		✓						✓
	Trust in physician [52]								
**Mental health and risk factors**									
	BINI^a^		✓						
	Depression (CES-D^b^) [53]		✓						✓
	AUDIT-C^c^ [54]		✓						
	Cocaine use disorder [55]		✓						
	Substance use disorder		✓						
	ASI-Lite^d^ to assess cocaine use, polysubstance use, and severity [56,57]		✓			✓	✓	✓	✓
	Timeline follow back [58]								
	LifeWindows (LW-IMB-AAQ^e^) questionnaire to measure the information, motivation, and behavioral constructs of adherence [59,60]		✓						
**Adherence**									
	Visual analog scale [61,62]		✓			✓	✓	✓	✓
	Wilson adherence scale [63]		✓			✓	✓	✓	✓
	Barriers to taking medication		✓					✓	
**Technology perceptions**									
	Technology acceptance [64]		✓			✓		✓	
	Credibility of SMS text message source [65]					✓		✓	
	Attitudes toward text and study							✓	
	**Geolocation and social network**								
	Activity space					✓		✓	
	Social network					✓		✓	
	Social network designee		✓						
	Smartphone survey					✓	✓	✓	✓
	Phone call history					✓	✓	✓	✓
**Payments (US $)**									
	Research interviews	N/A^f^	30	N/A	N/A	30	20	30	20
	Return of the devices (smartphone and pillbox)	N/A	N/A	N/A	N/A	N/A	N/A	N/A	90
	Weekly smartphone surveys	N/A	N/A	N/A	N/A	N/A	N/A	N/A	80
	Incentive to social network designee	N/A	N/A	N/A	N/A	N/A	N/A	N/A	20

^a^BINI: Brief Inventory of Neurocognitive Impairment.

^b^CES-D: Center for Epidemiologic Studies–Depression.

^c^AUDIT-C: Alcohol Use Disorders Identification Test–Concise.

^d^ASI-Lite: Addiction Severity Index–Lite.

^e^LW-IMB-AAQ: Life Windows Information Motivation Behavioral skills ART Adherence Questionnaire.

^f^N/A: not applicable.

#### Screening

The screening instrument included questions regarding HIV status, health insurance, current prescription of ART, social designees, and ability and willingness to use a smart pillbox and smartphone for the duration of the study. This instrument determined the eligibility of a participant.

#### Baseline

The research staff completed the baseline survey after participants provided consent and a signed medical release of information for self-reported HIV status and HIV treatment regimen. Neurocognitive impairment (NCI) was assessed at baseline before assigning participants to the study arms. In addition, questions regarding their social networks (requested for arm D) were also administered at baseline. Phlebotomy measures using Quest Diagnostics were also conducted at baseline to conduct tests that included HIV-1 RNA testing (Amplicor 1.5, range 50-750,000 copies/mL), CD4 lymphocyte count using flow cytometry, and HIV genotypic mutations (if VL>500 copies/mL).

#### Weekly Survey

During the intervention period (12 weeks), participants completed a short survey per week using the *CommCare* app on their smartphones. *CommCare* is a survey platform designed specifically for mobile devices. The survey had a multiple-choice format with the questions regarding cocaine use in the past 7 days, triggers of cocaine use, protected and unprotected sexual intercourse following cocaine use, ART adherence in the past 7 days, and reasons for nonadherence. The research team sent an automated SMS text message reminder to complete the survey.

#### Monthly Research Interviews

A total of 4 monthly interviews took place at weeks 4, 8, 12, and 16, the latter of which was the postintervention interview. At the week 8 follow-up, participants also completed a set of questions on the acceptability and feasibility of the study components using the Technology Acceptance Model. The questions were about the ease of use and usefulness of smart pillboxes and smartphones, attitude toward adherence-related SMS text messages, and perceived credibility of those messages. For participants in arm D of the RCT, there was an additional instrument measuring the acceptability and receptivity of the social network intervention.

#### SMS Text Messages

SMS text message data were collected from arm D participants at weeks 4, 8, and 12. A total of 4 SMS text messages (1 SMS text message per week) sent by the social designee to the participant were downloaded from participants’ phones by a research staff member. These messages were then saved in a secure and password-protected drive at X University’s Health Insurance Portability and Accountability Act–compliant server.

#### Study Completion and Device Recycling

At the week 16 interview, study participants were asked to return their pillbox and their smartphone. Incentives were built in for device returns (Table 1). The study team disinfected the phones and boxes and applied a factory reset to remove all the saved information from the prior user.

#### Payments

All participants were given payments in the form of gift cards for their time and participation, following the guidelines of our IRB. Participants were paid for monthly interviews, answering the weekly smartphone survey, and returning the smart pillboxes and smartphones at the end of the study. Refer to Table 1 for a description of the payments.

### Analytical Plan

#### Primary Outcomes

The primary outcomes of this study were ART adherence and persistence, measured as a composite score ranging from 0% to 100%, and as the number of continuous days of taking ART without a 7-day (or 3-day) gap [66,67], respectively.

#### Secondary Outcomes

Outcome analyses will be conducted for (1) drug outcomes (cocaine use), (2) HIV treatment outcomes (VL<20 copies/mL; change in CD4+ T lymphocytes), and (3) ART-related information, motivation, and behavioral skills constructs. The general framework for discerning differences between study conditions on the main outcome variables will be to perform a 4 (intervention condition)×3 (repeated follow-up assessments) mixed design analysis by entering preintervention scores as covariates and additional theoretically and empirically relevant variables (such as baseline mental status). For variables that are approximately normally distributed, we will use multivariate analyses of covariance, with significant multivariate tests with Bonferroni correction followed by subsequent analyses of covariance. Variables that violate distributional assumptions of normality will be analyzed using generalized linear model procedures, which enable the use of nonparametric functions such as non–Gaussian error models. On the basis of a review of the literature, the generalized linear model is the most appropriate approach for variables with strong positive skew, such as adherence [63,68].

### Ethical Oversight

The IRB at Yale University reviewed and approved all study procedures (IRB:1508016342). Additional protections were provided by the National Institute of Health, which issued a certificate of confidentiality.

## Results

### Sample Size

**Figure 3:**
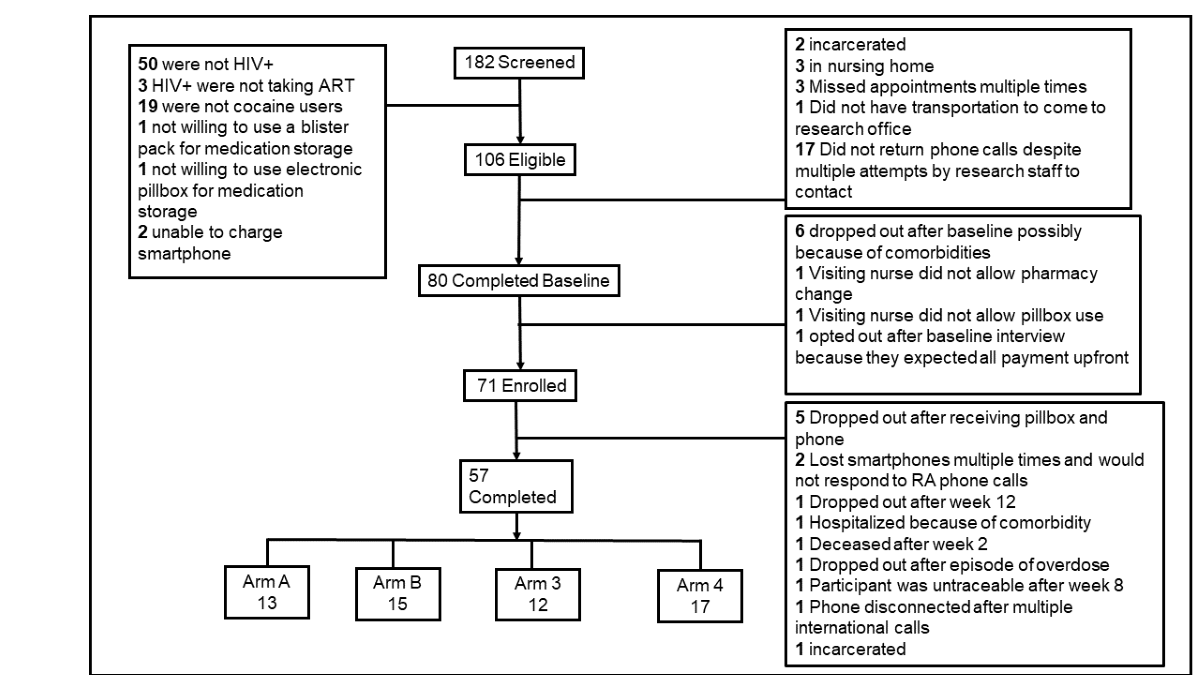
Study flow of participants. ART: antiretroviral therapy; RA: research assistant.

On the basis of a review of pilot studies that have ranged in sample size from 12 per group [69-71] to 9% to 50% [72,73] of a large RCT’s sample size (typically 20-30 per arm), we aimed to enroll 20 participants for each of the arms for a total of 80 participants. The final enrollment was 71 participants, of whom 57 (80%) completed the study. Figure 3 shows a complete CONSORT (Consolidated Standards of Reporting Trials) diagram. This was deemed appropriate for obtaining preliminary findings and optimizing a large-scale RCT for future studies [74].

### Randomization

Participants were assigned to their arms after completing the baseline interview and before receiving ART medication, a pillbox, and a smartphone. Owing to the time required in acquiring permission to change pharmacy and receive medication for 4 weeks from the pharmacy, there was a time-lapse between baseline interview and actual start time of the study, termed *preparation time* (Table 1). During the preparation time, participants were randomized to their respective arms, the pillbox communicative features were set to the requirements of the study arms, and the smartphones were set up with the survey (*CommCare*) and the SMS text message (*SMS Back-Up and Restore*) apps.

We followed a systematic sequence of assigning participants to the study arms—the first participant to arm A, second to arm B, third to arm C, and fourth to arm D; this sequence was repeated until the desired sample size was reached. In addition, during the baseline interview, questions related to the NCI were administered using the Brief Inventory of Neurocognitive Impairment questionnaire [75,76]. As it is well-documented that individuals with underlying mental illness experience worse outcomes, we stratified participants based on the presence or absence of NCI using 155 out of 228 as the cutoff point [75,76]. Consecutive participants with high NCI were placed in different arms. The data are currently undergoing analysis, and the manuscript is being developed for publication in early 2022.

## Discussion

### Study Challenges and Adaptations

#### Smart Pillbox

The research team chose the Health Insurance Portability and Accountability Act–compliant TowerView Health electronic pillbox because of its ability to record adherence data in real time and send automated reminders to a participant’s smartphone via SMS text messages. The *smart* features made this particular device more useful than frequently used pillboxes or bottles such as Glow Caps, MEMS Caps, or Wisepill in adherence research. As a start-up company based in Philadelphia, TowerView Health seemed to be the right choice for the study of the aforementioned reasons and for their active adherence research [77]. However, because of the unstable nature of start-up companies, this study experienced challenges worth noting.

First, the pillbox required a customized medication blister pack that would fit easily in the box. The local pharmacies at our study sites were not equipped with a printer to deliver these blister packs, creating the first barrier to adopting this pillbox. However, a pharmacy that had the capability to produce the customized blister packs required for the pillboxes was identified, located approximately 30 miles from the research site. The research team, along with TowerView Health, established a memorandum of understanding with the pharmacy that it would deliver participants’ HIV medication after receiving the prescription from the health care provider. This required participants to change their pharmacy for HIV medication for the duration of the study, which sometimes contributed to complications for the timely delivery of medication.

Second, some devices had technical issues similar to any other cellular device with wireless capabilities, such as loss of connection or cellular towers not responding. Our team worked diligently with the company’s engineering department and our participants to resolve such technical issues. One such incident, where pillboxes were not connected to the server because of negligence from the company’s side, led to data loss. Our research team was able to address this data loss by replacing faulty pillboxes with new devices and extending the study period for the affected participants to compensate for the data loss. We reached this solution by fully disclosing the errors and discussing the serious consequences on the study outcomes with our participants and the IRB. With IRB approval and full consent of the affected participants, we were able to resume regular intervention delivery and data collection.

Third, toward the end of Project SMART, when we were 8 participants away from reaching our target enrollment, TowerView Health informed us that their company had ceased to exist because of their inability to find investors. This was an unexpected challenge for our team; nevertheless, we addressed this issue by introducing a new method for the collection of adherence data for the last 8 participants. We extended the traditional and well-accepted pill count method of adherence to a picture format, whereby participants uploaded pictures of their ART pills in a blister pack on a weekly basis [78,79]. The collected pictures will allow us to count the pills taken in a given week and calculate the necessary adherence metrics based on prescribed dosages and time elapsed from the last refill [78-80]. Although we lost the real-time adherence data collected by the electronic pillboxes, the picture pill count provides an appropriate alternative to other more invasive and labor-intensive methods of measuring adherence.

#### Smartphones

Most participants were able to return their smartphones at the end of the study, which was then factory reset and reused for other study participants. However, some (9/57, 16%) participants reported losing or damaging their phones, such that phones had to be replaced. mHealth studies with incentives have reported a similar proportion of returned devices with return incentives. This is encouraging as the return rate among people with multiple challenges because of cocaine use is comparable with that of other populations.

The use of prepared Samsung phones for the participants provided numerous lessons for future digital health interventions. Each participant phone needed 2 separate apps (*CommCare* and *SMS Back-Up and Restore*) downloaded from the Google Play Store installed before participant enrollment. To ensure the consistency of the mobile phone hardware and software provided to each participant, the research team needed to register a new Google account on each Samsung device for each new participant. To prepare batches of phones with separate Google accounts, the research team needed to validate each new account by receiving an SMS text message on their personal phones. At the beginning of the study, Google account permissions allowed a single phone number to validate numerous new accounts. However, midway through the study, Google enacted more stringent account validation protocols, and the research team could not set up numerous accounts using 1 phone number. Thus, the creation of Google accounts to set up participant devices became a significant bottleneck for the project. The research team overcame this problem by using multiple phones to set up the accounts.

#### Time Length for Cocaine Use

One of the initial inclusion criteria was cocaine use in the past 30 days. This criterion was limiting, as most of the eligible participants reported using it infrequently or trying to quit. Hence, to address low recruitment, the study was adapted to first open up to people with HIV who had used cocaine in the past 60 days and then to people who had *ever used* to reach the target sample size, which was approved by the IRB.

#### Demands of the Study

Project SMART was conducted among people with HIV who were active cocaine users. Although most participants were able to adhere to the study protocol of using the electronic pillbox, using their smartphones to complete weekly surveys, and completing in-person interviews at the study site, almost one-fifth were unable to complete the study (14/71, 20%). The reasons for dropping out of the study were (1) comorbidities such as cancer and heart conditions, which required an extended period of hospitalization, affecting the ability to use the pillbox and participate in the study; (2) incarceration because of violent behavior while using illicit substances; (3) inability to answer phone calls and reminders for in-person interviews, leading to regularly missed in-person interviews; and (4) not being able to receive permission from nurses at the community homes to use medication adherence devices.

The RCT was a complex study that placed multiple demands on a population that already experienced constant instabilities, including insecurities related to health, social relations, or living conditions, because of drug use and other marginalized identities. The components of the study added to the burdens of living, including traveling to the research site, participating 4 times in an hour-long interview, learning how to use new technology, and completing multiple tasks throughout the period of the study. These demands created additional challenges for our participants, which resulted in a high dropout rate. Hence, in the future, similar mHealth interventions should aim to minimize these demands to reduce the burden on marginalized participants and maintain retention in the study.

This study lends itself to the emerging theoretical work on complex health intervention frameworks [81]. Complex health interventions are characterized by the complexity of the intervention at the following levels: the design of the intervention, the people involved (those who implement as well as those to receive it), at the organization and workforce, and at the outcome level. The 2 important constructs of this framework are the *core function* and *forms* of the intervention. *Core function* refers to the main purpose or goal that the intervention seeks to achieve, and *form* includes the strategies or activities of the intervention to achieve those goals [81]. The complex health intervention framework, which was based on research on patient-centered medical home interventions, argues that the core function of health interventions can be achieved through forms, which may vary greatly based on the cultural and contextual needs of the individuals that they serve. Hence, complex health interventions will change based on the context-specific needs of the patients or populations they serve as well as those who implement the intervention [81]. In this study, we acknowledge that the electronic pillbox was designed for individuals who have greater stability and fewer comorbidities than individuals with HIV who use cocaine. Therefore, future interventions should account for the complex and changing needs of this population, their health care providers (eg, nurses at community homes), and other characteristics in the early stages of the implementation and adapt the services to the changing demands of the intervention. Although this study was able to adjust the intervention in the latter part of the study by replacing the electronic pillbox with a manual blister package, earlier assessment and adaptation would have better facilitated the core function. In addition, the framework suggests that researchers should develop a *function and form matrix* in anticipation of challenges they may face in implementing complex interventions [81]. Hence, in the future, researchers should create a framework with the core functions of the interventions and possible adaptations of the forms to be prepared for the complexities that could arise at the various levels of the intervention.

### Conclusions

This paper provides detailed information about an RCT involving an mHealth intervention to improve adherence to ART for people with HIV who use cocaine. The 4-armed RCT with a control group sought to assess the feasibility and acceptability of technology interventions such as smart pillboxes and smartphones among at-risk, vulnerable populations, for whom currently the only effective adherence intervention is directly administered ART, which is expensive and labor intensive. The pilot study described here presents the different components of a technology-based intervention that can provide options for scaled-back strategies, both in terms of cost and personnel, as well as highlighting some challenges and adaptations required to consider when planning for a large-scale study.

## Appendix

Multimedia Appendix 1 Peer review report from the Behavioral and Social Consequences of HIV/AIDS Study Section - Center for Scientific Review (National Institutes of Health).

## Abbreviations

ART: antiretroviral therapy
CONSORT: Consolidated Standards of Reporting Trials
IRB: institutional review boardmHealth: mobile healthMTM: Motivation Technology Model
NCI: neurocognitive impairment
RCT: randomized controlled trial
VL: viral load
