# Correction: mHealth Intervention to Improve Treatment Outcomes Among People With HIV Who Use Cocaine: Protocol for a Pilot Randomized Controlled Trial

**DOI:** 10.2196/37925

**Published:** 2022-04-07

**Authors:** Yerina S Ranjit, Archana Krishnan, Debarchana Ghosh, Claire Cravero, Xin Zhou, Frederick L Altice

**Affiliations:** 1 Department of Communication University of Missouri Columbia, MO United States; 2 Department of Communication University at Albany State University of New York Albany, NY United States; 3 Department of Geography University of Connecticut Storrs, CT United States; 4 Harvard T H Chan School of Public Health Harvard University Boston, MA United States; 5 Department of Internal Medicine Section of Infectious Diseases Yale University New Haven, CT United States

In “mHealth Intervention to Improve Treatment Outcomes Among People With HIV Who Use Cocaine: Protocol for a Pilot Randomized Controlled Trial” (JMIR Res Protoc 2022;11(3):e28332) the authors noted two errors.

First, in the originally published article the affiliation for author Archana Krishnan appeared as follows:

Department of Social Scienes, University at Albany, State University of New York, Albany, NY, United States

This has been corrected as follows:

Department of Communication, University at Albany, State University of New York, NY, United States

Second, in the originally published article a footnote appeared below the affiliations as follows:

*all authors contributed equally

This was deleted in the correction, as it is not applicable.

The correction will appear in the online version of the paper on the JMIR Publications website on April 7, 2022, together with the publication of this correction notice. Because this was made after submission to PubMed, PubMed Central, and other full-text repositories, the corrected article has also been resubmitted to those repositories.

