# Supplementary material for: mHealth Intervention to Improve Treatment Outcomes Among People With HIV Who Use Cocaine: Protocol for a Pilot Randomized Controlled Trial
Source: JMIR Res Protoc. 2022 Mar 7;11(3):e28332. doi: 10.2196/28332 (PMC8938831; doi:10.2196/28332)
Supplement: Multimedia Appendix 1 [file resprot_v11i3e28332_app1.pdf]

SUMMARY STATEMENT
(Privileged Communication)

PROGRAM CONTACT:
Will Aklin
301-443-3207
aklinwm@mail.nih.gov

Release Date: **07/29/2015**

Application Number: **1 R21 DA039842-01A1**

Principal Investigator

ALTICE, FREDERICK LEWIS MD

Applicant Organization: YALE UNIVERSITY

Review Group: **BSCH**
Behavioral and Social Consequences of HIV/AIDS Study Section
AIDS - EXP. REV.

Meeting Date: **07/09/2015**
Council: **OCT 2015**
Requested Start: **09/01/2015**
RFA/PA: **PA14-181**
PCC: **CX/WMA**

Project Title: **Improving Antiretroviral Adherence and Persistence using mHealth Tools in HIV-infected Cocaine Users**
SRG Action: **Impact Score: 34**
Next Steps: **Visit http://grants.nih.gov/grants/next_steps.htm**
Human Subjects: **30-Human subjects involved - Certified, no SRG concerns**
Animal Subjects: **10-No live vertebrate animals involved for competing appl.**
Gender: **1A-Both genders, scientifically acceptable**
Minority: **1A-Minorities and non-minorities, scientifically acceptable**
Children: **1A-Both Children and Adults, scientifically acceptable**
Clinical Research - not NIH-defined Phase III Trial

Project Year	Direct Costs Requested	Estimated Total Cost
1	125,000	208,125
2	150,000	249,750
TOTAL	275,000	457,875

ADMINISTRATIVE BUDGET NOTE: The budget shown is the requested budget and has not been adjusted to reflect any recommendations made by reviewers. If an award is planned, the costs will be calculated by Institute grants management staff based on the recommendations outlined below in the COMMITTEE BUDGET RECOMMENDATIONS section.

1R21DA039842-01A1 ALTICE, FREDERICK

RESUME AND SUMMARY OF DISCUSSION: This application proposes to first conduct focus groups with persons living with HIV that also have cocaine use disorders (CUDs) and their healthcare providers to identify the barriers and facilitators to the implementation of mHealth interventions. This information will be used to design a 12-week RCT for HIV+ individuals with CUDs that will seek to examine the impact of mHealth tools and automated feedback vs. automated feedback + clinician feedback on ART adherence, viral suppression, cocaine use and retention in care. Adherence and retention in care are important issues and an intervention that addresses these issues using mobile devices has the potential to have a significant public health impact. The study team is very strong and has the expertise to carry out the proposed study. The applicant has been responsive to the concerns raised in the prior review of this application. The qualitative assessment was significantly improved, the RCT was described more fully and some other minor weaknesses were addressed. The inclusion of the MacArthur Competence Assessment Tool was an improvement as was the stratification for an Axis 1 depressive disorder. However, these changes did not fully address the concern about the impact of cognitive impairment on the proposed intervention. This concern and some other remaining minor concerns served to limit the committee's otherwise much improved enthusiasm for this resubmission.

DESCRIPTION (provided by applicant): Antiretroviral therapy (ART) is effective at lowering HIV-related mortality and reducing transmission among people living with HIV (PLH). Substance use disorders, especially cocaine use disorders (CUDs) greatly reduce ART adherence and persistence on treatment. Because stimulants like CUDs are not amenable to medication-assisted therapy and directly administered antiretroviral therapy is costly and unwieldy in most settings, evidence-based interventions are urgently needed to improve ART adherence and persistence in PLWH with co-occurring CUDs. International guidelines now suggest that the next generation of adherence interventions will need to be scaled back in terms of cost and personnel. Mobile technologies can provide innovative, efficacious and cost-effective strategies to improve ART adherence and optimize HIV treatment outcomes. Such technologies have great applicability in resource-limited settings due to their low cost and ubiquitous nature. Mobile health (mHealth) tools have been shown to improve adherence in patients with various chronic conditions including diabetes, tuberculosis, and HIV; however currently, no published research exists on the impact of mHealth interventions on adherence among PLH and CUDs. Thus, in response to PA- 14-181, "mHealth Tools for Individuals with Chronic Conditions to Promote Effective Patient-Provider Communication, Adherence to Treatment and Self-Management", we intend to first conduct qualitative research to assess the acceptability and feasibility of implementing mHealth interventions followed by a pilot feasibility study to examine the effect of mHealth tools on ART adherence. This study is particularly innovative as it proposes to use mHealth intervention with various types of feedback, on PLH with co-occurring CUDs – a group with problematic ART adherence and persistence. The specific aims are: (1) to conduct qualitative assessments using focus groups of PLH who use cocaine and healthcare providers that will assess the acceptability, feasibility, facilitators and barriers of implementing mHealth interventions; and will aid in developing the final design and content of both automated and clinician feedback in preparation for designing a pilot feasibility study; and (2) to conduct a 12-week pilot feasibility RCT among PLH with co-occurring CUDs that will examine the impact of mHealth tools (cellular-enabled smart pill boxes and cell phones) and feedback (no feedback vs. automated feedback vs. automated + clinician feedback) on primary (ART adherence and persistence) and secondary outcomes (HIV viral suppression, cocaine use, retention in HIV care). Findings from this pilot study will help in refining the intervention, developing a research protocol for a future R01 and providing data for a larger-scale RCT. This research will have widespread implications for the use of mHealth tools as an innovative adherence strategy in a population with profound health disparities.

PUBLIC HEALTH RELEVANCE: This project seeks to examine the impact of mHealth tools on ART adherence and persistence among HIV+ cocaine users through qualitative assessments and a 12-week pilot feasibility RCT. Qualitative assessments will consist of focus groups among cocaine users and healthcare providers that will evaluate the acceptability, feasibility, facilitators and barriers of implementing mHealth interventions. The pilot feasibility RCT will examine the impact of mHealth tools (cellular-enabled smart pill boxes and cell phones) and feedback (no feedback vs. automated feedback vs. automated + clinician feedback) on ART adherence and persistence, HIV viral suppression, cocaine use, and retention in HIV care.

CRITIQUE 1:

Significance: 2
Investigator(s): 1
Innovation: 1
Approach: 7
Environment: 2

Overall Impact: This is a moderately responsive revision of an application seeking support for a trial of a multi-component intervention to improve medication adherence in HIV-infected individuals with cocaine use disorders. While the problem to be addressed is highly significant, the study's underlying theoretical rationale is difficult to discern and important details about how the study intervention will be delivered are not clear, substantially reducing this study's likely overall impact.

1. Significance:

Strengths

- The study addresses adherence in an important population from a public health perspective.

Weaknesses

- None

2. Investigator(s):

Strengths

- The investigative team is strong.

Weaknesses

- None

3. Innovation:

Strengths

- The use of the MedSignals device in combination with IVR technology is innovative.

Weaknesses

- Study design and implementation follows a standard approach.

4. Approach:

Strengths

- The proposed study will use the investigative team's well-established capabilities in developing an intervention that addresses adherence in an important groups and that has the potential to be cost-effective.

Weaknesses

- Inclusion criteria state language may be either English or Spanish. It's not clear how persons whose preferred language is Spanish would participate in the planned focus groups or the intervention.
- Focus groups will be completed with participants who report cocaine use in the past 30 days, while the intervention study will focus on those with cocaine use disorders. As it is possible that casual users may differ from those with more clear impairment, a better rationale for this strategy would strengthen the application.
- It is still difficult to discern how the MedSignals device will provide individualized feedback to study participants on their level of adherence. The exact nature of clinician-provided feedback to individuals is not clearly explained beyond the investigators' suggestion that it would be related to the IMB model.
- The investigators have been responsive to previous reviewers' concerns about the relevance of the proposed intervention to the IMB model, but it is still not evident how automated reminding maps onto IMB elements, beyond its ability to address a skill deficit (remembering to take medication). If, as the investigators suggest, the automated and clinician feedback will incrementally improve adherence skills then it would be useful to better understand how the intervention will do this.
- Given the equivocal results of the LifeWindows study, it would be helpful to understand how the intervention will be made more effective by using its model. The relevance of the LifeWindows materials included in the Appendix to the planned Medsignals/IVR/clinician intervention is not clear.
- How reminding is synergistic with the elements of the IMB model is not clear.
- Given the extensive body of research on the influence of cognition on adherence, failing to take cognitive status into account is a significant weakness.

5. Environment:

Strengths

- The environment in which the study would be completed is excellent.

Weaknesses

- None

Protections for Human Subjects:

Acceptable Risks and/or Adequate Protections

Data and Safety Monitoring Plan (Applicable for Clinical Trials Only):

Acceptable

Inclusion of Women, Minorities and Children:

- Sex/Gender: Distribution justified scientifically
- Race/Ethnicity: Distribution justified scientifically
- Inclusion/Exclusion of Children under 21: Including ages < 21 justified scientifically

Vertebrate Animals:

Not Applicable (No Vertebrate Animals)

Resource Sharing Plans:

Not Applicable (No Relevant Resources)

Budget and Period of Support:

Recommend as Requested

CRITIQUE 2:

Significance: 1
Investigator(s): 2
Innovation: 1
Approach: 3
Environment: 1

Overall Impact: This experienced and highly productive team of investigators proposes to conduct qualitative research to assess the acceptability and feasibility of implementing mHealth interventions followed by a pilot feasibility study to examine the effect of mHealth tools on ART adherence in a population of HIV-positive individuals with cocaine use disorders – a group with problematic ART adherence and persistence. The proposal was responsive to prior critiques regarding study design, measurement and analysis, and has high potential for a significant public health impact given the paucity of available adherence interventions for the marginalized population under study.

1. Significance:

Strengths

- Targeting HIV-positive individuals with cocaine use disorders is highly significant given the well-established challenges in ART adherence and persistence in this population, and paucity of established, efficacious interventions to improve HIV care outcomes.

Weaknesses

- None noted.

2. Investigator(s):

Strengths

- PI Frederik Altice has considerable experience on “development and evaluation of innovative strategies to identify, link, treat and retain HIV-infected individuals in care who have underlying

substance use"—this is evidenced by his involvement with a number of RCTs to evaluate interventions to improve ARV adherence among HIV+ people who use drugs
- Substantial experience among other applicants (Krishnan; Copenhaver) in RCTs of interventions to improve adherence and other outcomes among substance users.
- The PI has experience in qualitative research, demonstrate by his role as PI on the project "Expanding Medication Assisted Therapies in Ukraine"
- History of previous collaboration between Atice, Copenhaver and Krishnan.

Weaknesses

- Given that 25% of the effort for this project comes from Dr. Shan-Estelle Brown (other personnel), a bit more detail about her research experience was expected. However, from what is provided, she seems to be well-suited to lead the qualitative component of this study given her background in Medical Anthropology and research focus on substance users' attitudes toward medical technologies.

3. Innovation:

Strengths

- While an mHealth initiative such as the one proposed is not necessarily innovative in and of itself, its application to a population with cocaine use disorders is unique, and may result in valuable guidance on future HIV care interventions in this population regardless of the outcome of the trial.

Weaknesses

- None noted.

4. Approach:

Strengths

- The scope, content and context of the proposal is an excellent fit with the R21 funding mechanism. Given the novelty of the intervention, it is absolutely appropriate and prudent to pilot test this form of intervention in the targeted patient population.
- The acknowledgement and inclusion of persistence as well as adherence as outcomes is a strength
- Acknowledgement of cost considerations - and the impact of these considerations on the scalability of any such interventions – is also a strength
- The investigators have provided a wealth of detail regarding study instruments and procedures – thus addressing the key weaknesses identified in prior submission
- The analysis plan for the pilot has now been sufficiently developed.

Weaknesses

- Given the emphasis on efficiency in the significance section, it is somewhat surprising that the investigators have not proposed a basic analysis to estimate the costs of delivering each intervention, or otherwise used the pilot to determine whether health care cost data collection is feasible in this population.

5. Environment:

Strengths

- This study is ideally situated. The environment is very strong and well suited to carry out the proposed study.

Weaknesses

- None noted.

Protections for Human Subjects:

Acceptable Risks and/or Adequate Protections

Data and Safety Monitoring Plan (Applicable for Clinical Trials Only):

Acceptable

Inclusion of Women, Minorities and Children:

- Sex/Gender: Distribution justified scientifically
- Race/Ethnicity: Distribution justified scientifically
- Inclusion/Exclusion of Children under 21: Including ages < 21 justified scientifically

Vertebrate Animals:

Not Applicable (No Vertebrate Animals)

Biohazards:

Not Applicable (No Biohazards)

Resubmission:

- This proposal was highly responsive to prior critiques, resulting in a very strong proposal.

Budget and Period of Support:

Recommend as Requested

CRITIQUE 3:

Significance: 1
Investigator(s): 1
Innovation: 2
Approach: 3
Environment: 1

Overall Impact: This resubmission of an R21 application proposes to determine the acceptability, study design, and implementation procedures for carrying out an RCT built upon patient-mHealth-clinician relationship for improving ART adherence and persistence among PLH with cocaine use

disorders (CUDs). Qualitative assessment phase utilizing feedback from patients with CUDs and healthcare providers will inform the pilot trial and feedback elements. Use of mHealth devices and real-time feedback – mapped onto the IMB model – are proposed as the primary tools of the intervention. The investigative team is strong, methods are clear, and the environment is well suited to conduct the study. Minor issues remain in the approach but are quite addressable.

1. Significance:

Strengths

- Persons with cocaine use disorders continue to be at high risk for poor medication adherence; approaches to reduce their risk has implications for their own health as well as reducing HIV transmission.
- Interventions that can communicate in “real time” may hold promise for high risk groups.

Weaknesses

- None noted.

2. Investigator(s):

Strengths

- The investigative team is strong and has relevant expertise in the various components of the study.

Weaknesses

- None noted.

3. Innovation:

Strengths

- Although the IMB itself is not innovative, its application to mHealth interventions and the substance using population is an innovative use of the model.

Weaknesses

- None noted.

4. Approach:

Strengths

- Qualitative methods are well defined.
- The intervention elements are mapped on to the theoretical framework.
- The inclusion of the MacArthur Competence Assessment Tool is a strength
- Use of the focus groups to identify how messages should be tailored will improve the relevance of messages.
- Prior experience and data showing that unstably housed individuals can participate in mHealth-type intervention studies supports the methods proposed herein.

Weaknesses

- Study timeline for both qualitative and pilot feasibility elements may be ambitious.

- A feasibility study does not require sophisticated analysis plan (although it is assumed that the proposed models are to generate effect sizes for the future R01).
- Prior review noted that the study population is at risk for cognitive impairment. This resubmission attempts to address this concern via stratification of an Axis I Depressive Disorders. Although an important variable, this does not account for the presence of cognitive impairment that may dilute intervention effectiveness.

5. Environment:

Strengths

- The environment is excellent and well-suited to support the proposed study.

Weaknesses

- None noted.

Protections for Human Subjects:

Acceptable Risks and/or Adequate Protections

- Use of the MacArthur measure for competence is a particular strength as is inclusion of a psychiatrist and clear plan for referring persons with clinically significant depressive symptoms.

Data and Safety Monitoring Plan (Applicable for Clinical Trials Only):

Not Applicable (No Clinical Trials)

Inclusion of Women, Minorities and Children:

- Sex/Gender: Distribution justified scientifically
- Race/Ethnicity: Distribution justified scientifically
- Inclusion/Exclusion of Children under 21: Including ages < 21 justified scientifically

Vertebrate Animals:

Not Applicable (No Vertebrate Animals)

Biohazards:

Not Applicable (No Biohazards)

Resubmission:

- Applicants were mostly responsive to reviewer comments and have made important adjustments, justification, and clarifications.

Budget and Period of Support:

Recommend as Requested

THE FOLLOWING SECTIONS WERE PREPARED BY THE SCIENTIFIC REVIEW OFFICER TO SUMMARIZE THE OUTCOME OF DISCUSSIONS OF THE REVIEW COMMITTEE, OR REVIEWERS' WRITTEN CRITIQUES, ON THE FOLLOWING ISSUES:

PROTECTION OF HUMAN SUBJECTS (Resume): ACCEPTABLE

INCLUSION OF WOMEN PLAN (Resume): ACCEPTABLE

INCLUSION OF MINORITIES PLAN (Resume): ACCEPTABLE

INCLUSION OF CHILDREN PLAN (Resume): ACCEPTABLE

COMMITTEE BUDGET RECOMMENDATIONS: The budget was recommended as requested.

NIH has modified its policy regarding the receipt of resubmissions (amended applications). See Guide Notice NOT-OD-14-074 at http://grants.nih.gov/grants/guide/notice-files/NOT-OD-14-074.html. The impact/priority score is calculated after discussion of an application by averaging the overall scores (1-9) given by all voting reviewers on the committee and multiplying by 10. The criterion scores are submitted prior to the meeting by the individual reviewers assigned to an application, and are not discussed specifically at the review meeting or calculated into the overall impact score. Some applications also receive a percentile ranking. For details on the review process, see http://grants.nih.gov/grants/peer_review_process.htm#scoring.

MEETING ROSTER

Behavioral and Social Consequences of HIV/AIDS Study Section
AIDS and Related Research Integrated Review Group
CENTER FOR SCIENTIFIC REVIEW
BSCH
July 09, 2015 - July 10, 2015

CHAIRPERSON

SEAL, DAVID W, PHD
PROFESSOR
DEPARTMENT OF GLOBAL COMMUNITY HEALTH
AND BEHAVIORAL SCIENCES
SCHOOL OF PUBLIC HEALTH AND TROPICAL MEDICINE
TULANE UNIVERSITY
NEW ORLEANS, LA 70112

MEMBERS

BOGART, LAURA M, PHD
ASSOCIATE PROFESSOR
DEPARTMENT OF MEDICINE
HARVARD MEDICAL SCHOOL
BOSTON, MA 02215

BROWN, LARRY K, MD *
PROFESSOR
DEPARTMENT OF PSYCHIATRY AND HUMAN BEHAVIOR
SCHOOL OF MEDICINE
BROWN UNIVERSITY
PROVIDENCE, RI 02912

COOK, ROBERT L, MPH, MD
PROFESSOR
DEPARTMENT OF EPIDEMIOLOGY
UNIVERSITY OF FLORIDA
GAINESVILLE, FL 32610

CORSI, KAREN F, MPH, SCD
ASSOCIATE PROFESSOR
DEPARTMENT OF PSYCHIATRY
SCHOOL OF MEDICINE
UNIVERSITY OF COLORADO, DENVER
DENVER, CO 80262

CROPSEY, KAREN L, PSYD *
ASSOCIATE PROFESSOR
DEPARTMENT OF PSYCHIATRY
UNIVERSITY OF ALABAMA AT BIRMINGHAM
BIRMINGHAM, AL 35294

ESSIEN, EKERE JAMES, MD, DRPH *
PROFESSOR
DEPARTMENT OF CLINICAL SCIENCES
AND ADMINISTRATION
COLLEGE OF PHARMACY
UNIVERSITY OF HOUSTON
HOUSTON, TX 77030

GOLUB, SARIT A, PHD *
PROFESSOR
DEPARTMENT OF PSYCHOLOGY
HUNTER COLLEGE
NEW YORK, NY 10065

GORE-FELTON, CHERYL E, PHD *
PROFESSOR
DEPARTMENT OF PSYCHIATRY AND BEHAVIORAL
SCIENCES
SCHOOL OF MEDICINE
STANFORD UNIVERSITY
STANFORD, CA 94305

KUHNS, LISA MARY, PHD *
ASSISTANT PROFESSOR
DEPARTMENT OF PEDIATRICS
FEINBERG SCHOOL OF MEDICINE
NORTHWESTERN UNIVERSITY
CHICAGO, IL 60612

LEHMAN, WAYNE E K, PHD *
SENIOR RESEARCH SCIENTIST
INSTITUTE OF BEHAVIOIRAL RESEARCH
TEXAS CHRISTIAN UNIVERSITY
FORT WORTH, TX 76129

LI, XIAOMING , PHD
PROFESSOR
DEPARTMENT OF PEDIATRICS
SCHOOL OF MEDICINE
WAYNE STATE UNIVERSITY
DETROIT, MI 48201

MERCHANT, ROLAND C, MPH, SCD, MD
ASSOCIATE PROFESSOR
DEPARTMENT OF EMERGENCY MEDICINE
WARREN ALPERT MEDICAL SCHOOL
BROWN UNIVERSITY
PROVIDENCE, RI 02903

METSCH, LISA R, PHD *
PROFESSOR AND CHAIR
DEPARTMENT OF SOCIOMEDICAL SCIENCES
MAILMAN SCHOOL OF PUBLIC HEALTH
COLUMBIA UNIVERSITY
NEW YORK, NY 10032

MOSKOWITZ, JUDITH T, PHD *
PROFESSOR
DEPARTMENT OF MEDICAL SOCIAL SCIENCES
FEINBERG SCHOOL OF MEDICINE
NORTHWESTERN UNIVERSITY
CHICAGO, IL 60611

NOSYK, BOHDAN , PHD *
ASSOCIATE PROFESSOR AND ENDOWED CHAIR
ECONOMICS OF HIV/AIDS
FACULTY OF HEALTH SCIENCES
SIMON FRASER UNIVERSITY AND
BC CENTRE FOR EXCELLENCE IN HIV/AIDS
VANCOUVER, BC V6Z1Y6
CANADA

NYAMATHI, ADELINE M, BSN, PHD, FAAN *
DISTINGUISHED PROFESSOR
SCHOOL OF NURSING
UNIVERSITY OF CALIFORNIA, LOS ANGELES
LOS ANGELES, CA 90095

OWNBY, RAYMOND L, MD, PHD *
PROFESSOR AND CHAIR
DEPARTMENT OF PSYCHIATRY AND BEHAVIORAL MEDICINE
COLLEGE OF OSTEOPATHIC MEDICINE
NOVA SOUTHEASTERN UNIVERSITY
FORT LAUDERDALE, FL 33314

RAO, DEEPA , PHD *
ASSOCIATE PROFESSOR
DEPARTMENT OF GLOBAL HEALTH
SCHOOL OF MEDICINE
UNIVERSITY OF WASHINGTON
SEATTLE, WA 98104

REYNOLDS, NANCY R, PHD
PROFESSOR
SCHOOL OF NURSING
YALE UNIVERSITY
NEW HEAVEN, CT 06536

SANTOS, GLENN-MILO , PHD *
SENIOR RESEARCH SCIENTIST
CENTER FOR PUBLIC HEALTH RESEARCH BRANCH
SAN FRANCISCO DEPARTMENT OF PUBLIC HEALTH
SAN FRANCISCO, CA 94102

SORENSEN, JAMES L, PHD *
PROFESSOR
DEPARTMENT OF PSYCHIATRY
UNIVERSITY OF CALIFORNIA, SAN FRANCISCO
SAN FRANCISCO, CA 94110

STANTON, CASSANDRA A, PHD *
ASSISTANT PROFESSOR
DEPARTMENT OF ONCOLOGY
LOMBARDI COMPREHENSIVE CANCER CENTER
GEORGETOWN UNIVERSITY MEDICAL CENTER
WASHINGTON, DC 20007

TOBIN, KARIN E, PHD *
ASSOCIATE PROFESSOR
DEPARTMENT OF HEALTH, BEHAVIOR, AND SOCIETY
BLOOMBERG SCHOOL OF PUBLIC HEALTH
JOHNS HOPKINS UNIVERSITY
BALTIMORE, MD 21205

WALDROP-VALVERDE, DRENNA , PHD
ASSOCIATE PROFESSOR
NELL HODGSON WOODRUFF SCHOOL OF NURSING
EMORY UNIVERSITY
ATLANTA, GA 30322

YBARRA, MICHELE , PHD *
PRESIDENT AND RESEARCH DIRECTOR
CENTER FOR INNOVATIVE PUBLIC HEALTH RESEARCH
SANTA ANA, CA 92672

YOUNG, SEAN , PHD *
ASSISTANT PROFESSOR
CENTER FOR BEHAVIORAL AND ADDICTION MEDICINE
DEPARTMENT OF FAMILY MEDICINE
UNIVERSITY OF CALIFORNIA LOS ANGELES
LOS ANGELES, CA 90024

MAIL REVIEWER(S)

HALLFORS, DENISE DION, PHD
SENIOR RESEARCH SCIENTIST
MATERNAL AND CHILD HEALTH
PACIFIC INSTITUTE FOR RESEARCH AND EVALUATION
CHAPEL HILL, NC 27514

SCIENTIFIC REVIEW OFFICER

RUBERT, MARK P, PHD
SCIENTIFIC REVIEW OFFICER
CENTER FOR SCIENTIFIC REVIEW
NATIONAL INSTITUTES OF HEALTH
BETHESDA, MD 20892

EXTRAMURAL SUPPORT ASSISTANT

BOGLEY, DAVID M
LEAD EXTRAMURAL SUPPORT ASSISTANT
CENTER FOR SCIENTIFIC REVIEW
NATIONAL INSTITUTES OF HEALTH
BETHESDA, MD 20892

* Temporary Member. For grant applications, temporary members may participate in the entire meeting or may review only selected applications as needed.

Consultants are required to absent themselves from the room during the review of any application if their presence would constitute or appear to constitute a conflict of interest.
